# Supplementary material for: Forecasting the Pharmacological Mechanisms of Plumbago zeylanica and Solanum xanthocarpum in Diabetic Retinopathy Treatment: A Network Pharmacology, Molecular Docking, and Molecular Dynamics Simulation Study
Source: Biology (Basel). 2024 Sep 18;13(9):732. doi: 10.3390/biology13090732 (PMC11429473; doi:10.3390/biology13090732)
Supplement: Supplementary file 1 [file biology-13-00732-s001.zip › biology-3178719-supplementary.pdf]

Forecasting the Pharmacological Mechanisms of *Plumbago zeylanica* and *Solanum xanthocarpum* in Diabetic Retinopathy Treatment: A Network Pharmacology, Molecular Docking, and Molecular Dynamics Simulation Study

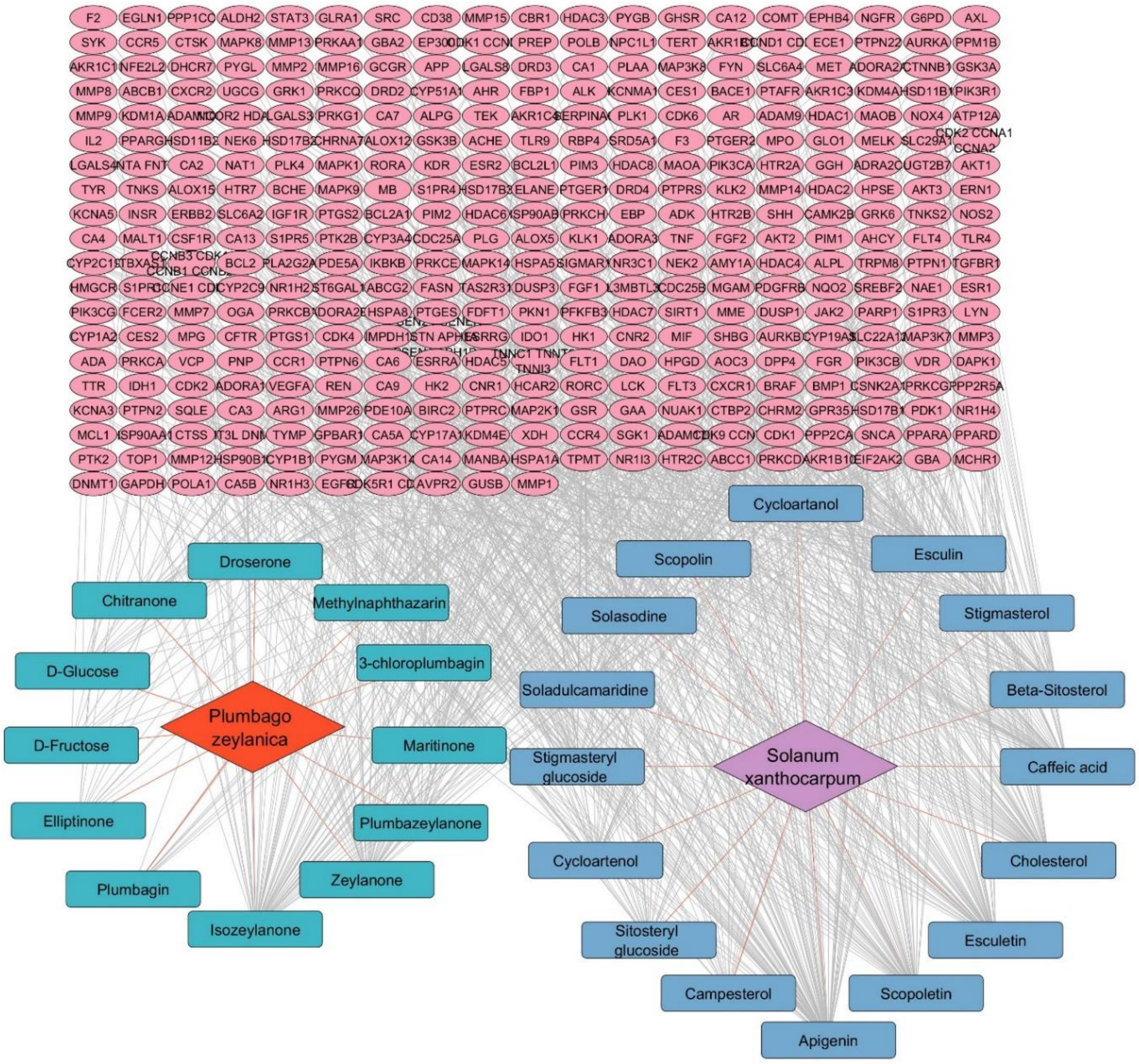

Figure S1. Phytoconstituent-related target network (Orange diamond node: PZ, Purple diamond node: SX, Green round rectangle node: PZ phytoconstituents, Blue round rectangle node: SX phytoconstituents, Pink elliptical node: Targets and edges show the interaction between two nodes)

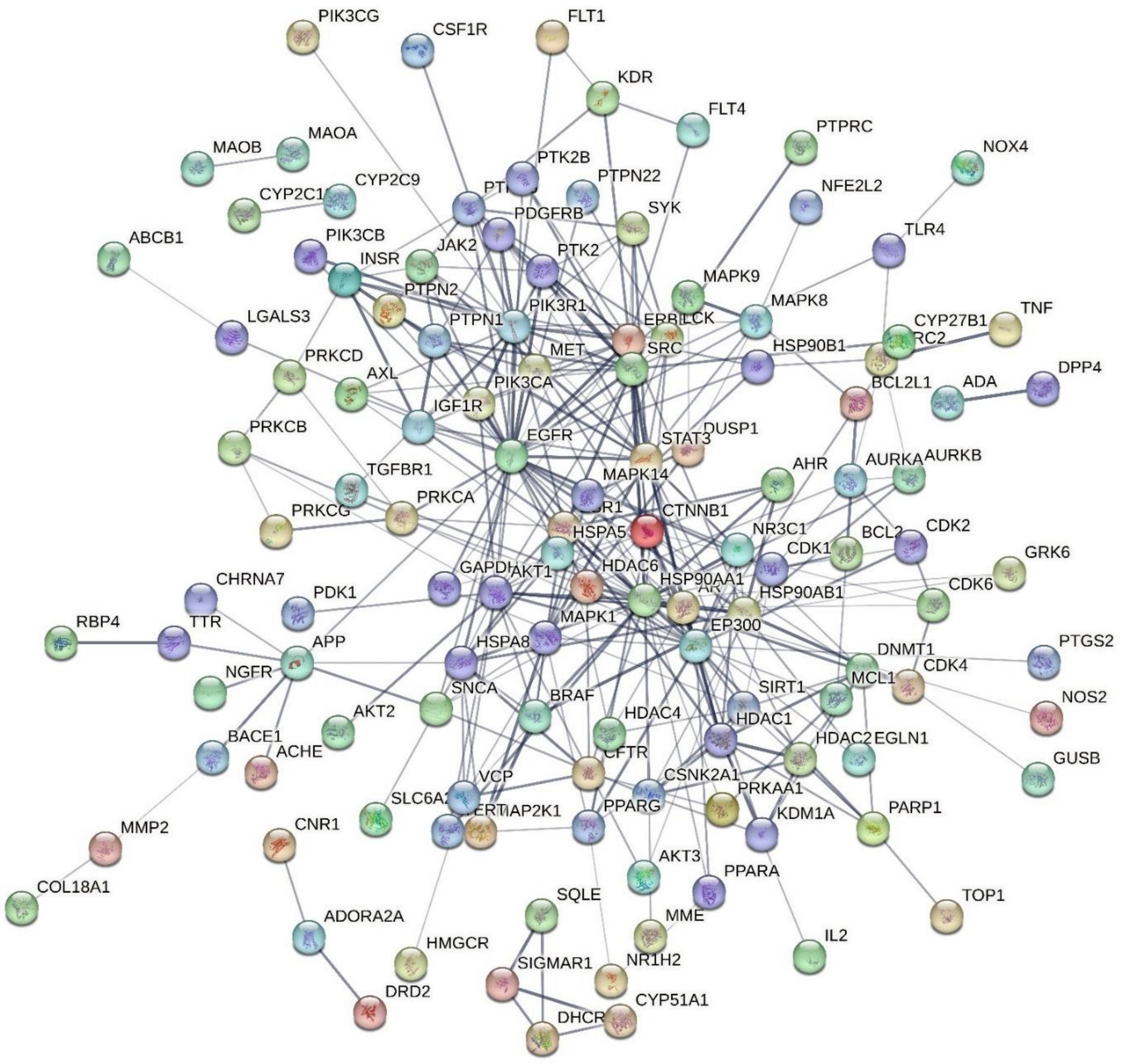

Figure S2. STRING obtained the PPI network (The nodes represent the target proteins and the edge represents the relation between the nodes: the darker the edge the more the node degree between two nodes and vice versa).

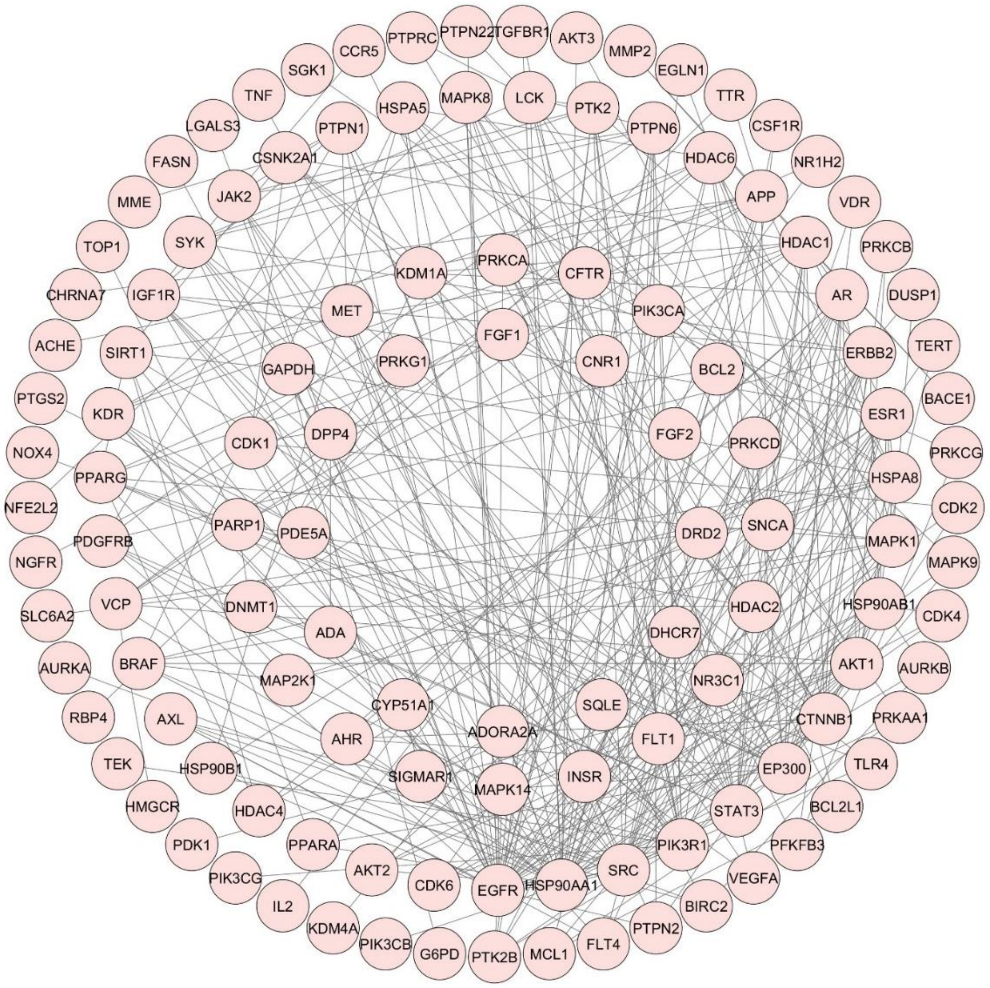

Figure S3. PPI network obtained from Cytoscape (Nodes represent receptor targets and edges represent their relation between two nodes)

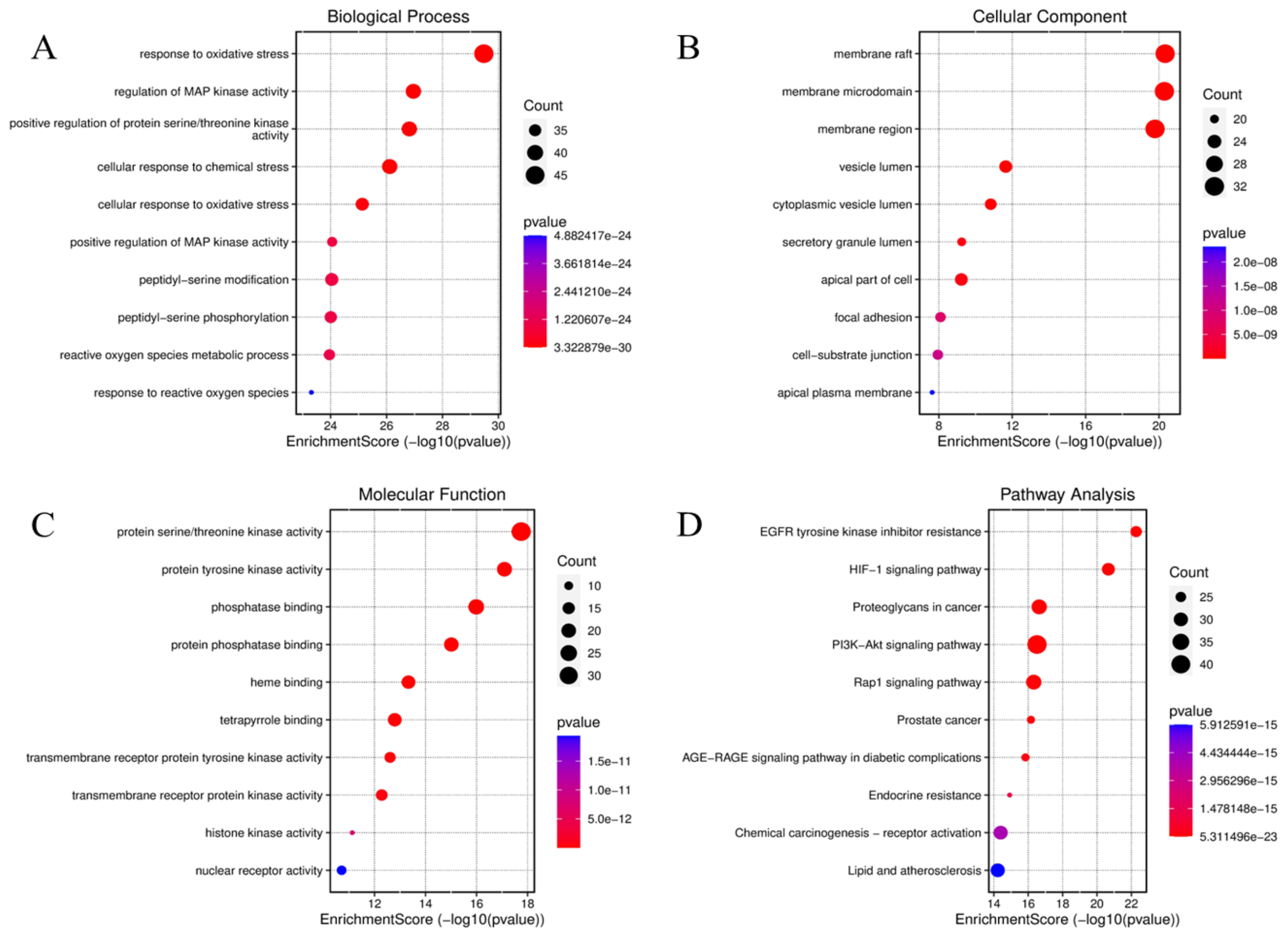

Figure S4. (A) shows the dot-plot diagram representing the enrichment of BPs; (B) displays the dot-plot diagram for the enrichment of CCs; (C) depicts the dot-plot diagram for the enrichment of MFs; (D) illustrates the dot-plot diagram for KEGG pathway enrichment analysis, where pathways are ranked by fold enrichment. In this diagram, the size of each dot corresponds to the number of genes, the x-axis indicates -logP values, and the color of the dots represents the p-value.

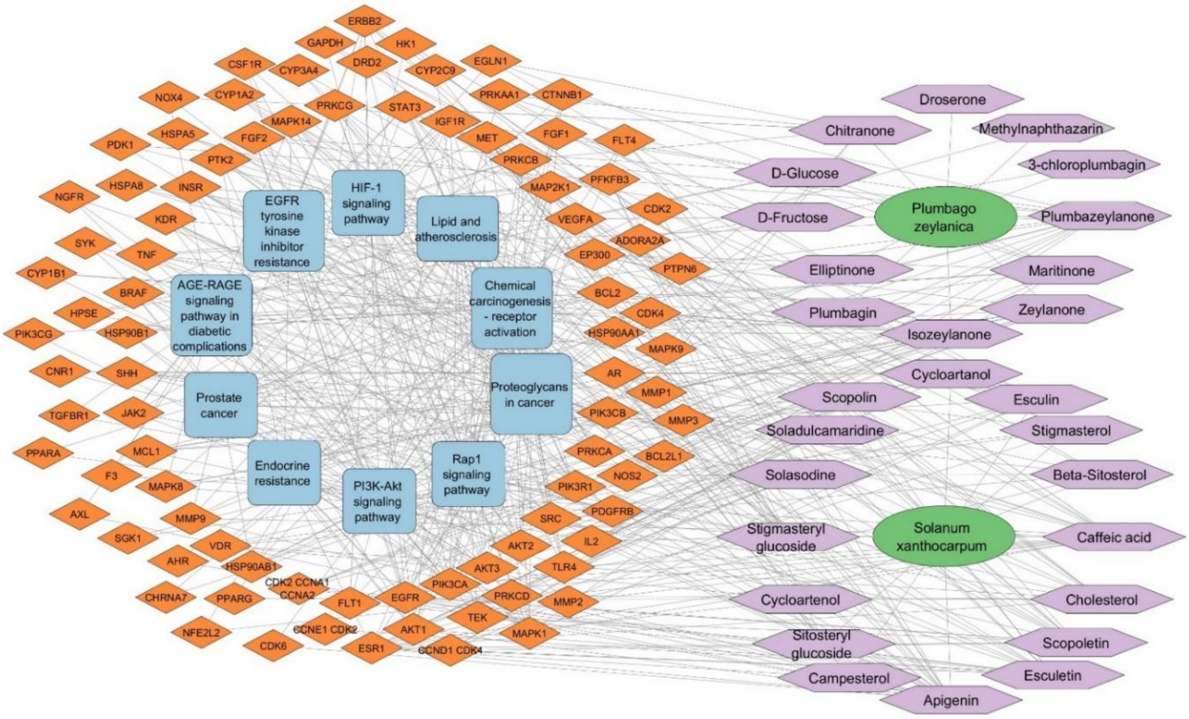

Figure S5. SX & PZ-Phytoconstituents-Targets-Pathways Network (Green elliptical nodes show SX & PZ, purple hexagon nodes show SX & PZ-phytoconstituents, orange diamond nodes show target proteins/genes, blue round rectangle nodes show pathways and edges show the interaction between two nodes)

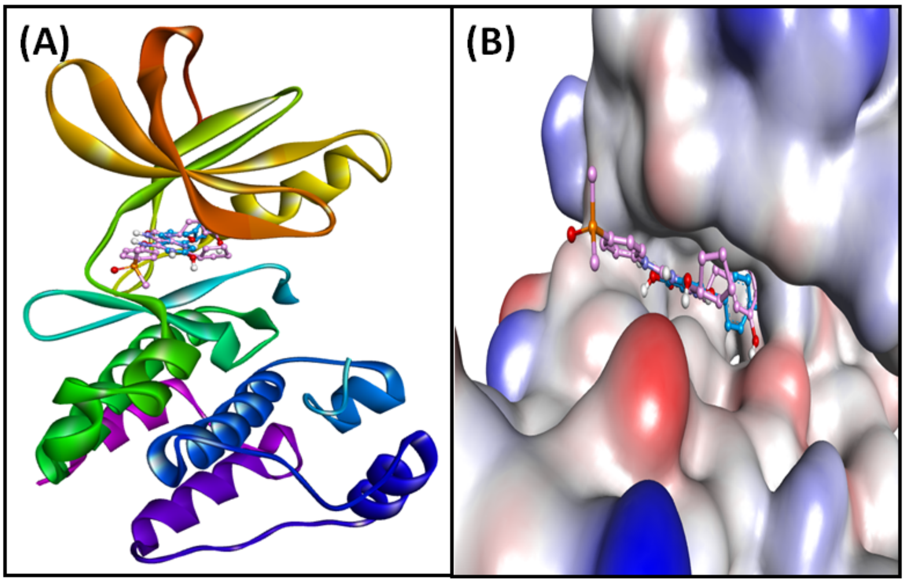

Figure S6. 3D image of SRC-Apigenin docked complex result comparing with AP23464 binding pocket region. (A: SRC-Apigenin/AP23464 docked complex, B: Allosteric binding pocket region SRC-Apigenin/AP23464 docked complex)

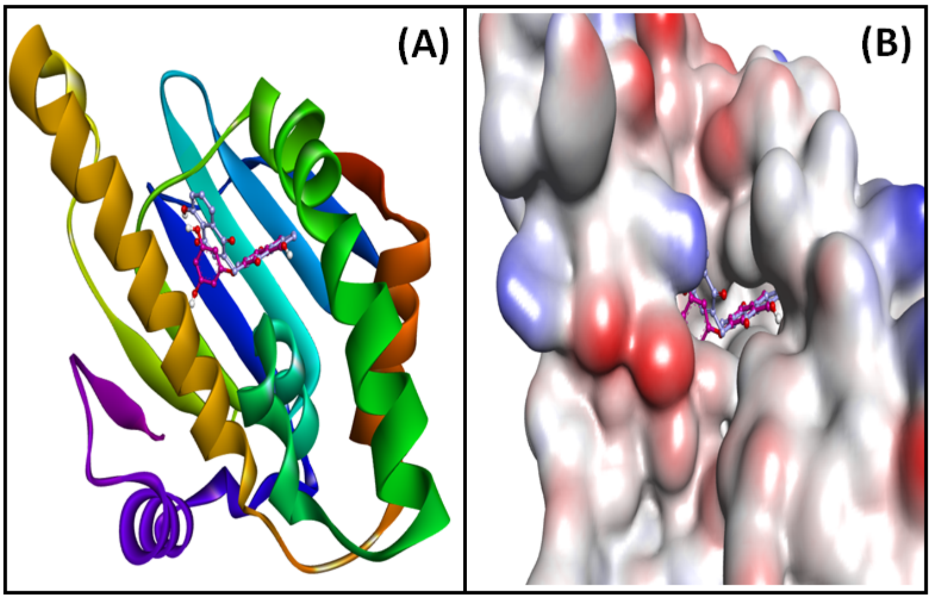

Figure S7. 3D image of HSP90AA1- Isozeylanone docked complex result comparing with NMS-E973 binding pocket region. (A: HSP90AA1- Isozeylanone /NMS-E973 docked complex, B: Allosteric binding pocket region HSP90AA1- Isozeylanone/NMS-E973 docked complex)

Table S1. Molecular interaction parameters for the binding of ligands to SRC.

Ligand	Donor-Acceptor pair	Distance (Å)	Type of Interaction
**Control* (AP23464)**	LIG:HN - MET341:O	2.8204	Conventional Hydrogen Bond
	LIG:H - MET341:O	2.2593	Conventional Hydrogen Bond
	LIG:C - GLU339:O	3.6546	Carbon Hydrogen Bond
	LYS295:HZ2 - LIG	3.3089	Pi-Cation (Electrostatic);Pi-Donor (Hydrogen Bond)
	LEU273:CD2 - LIG	3.6363	Hydrophobic (Pi-Sigma)
	THR338:CG2 - LIG	3.8464	Hydrophobic (Pi-Sigma)
	LEU393:CD2 - LIG	3.6740	Hydrophobic (Pi-Sigma)
	VAL281 - LIG	3.7542	Hydrophobic (Alkyl)
	LIG - LEU273	4.7349	Hydrophobic (Pi-Alkyl)
	LIG - VAL281	4.8529	Hydrophobic (Pi-Alkyl)
	LIG - ALA293	5.2965	Hydrophobic (Pi-Alkyl)
	LIG - LYS295	4.0378	Hydrophobic (Pi-Alkyl)
**Apigenin**	MET341:HN - LIG:O	2.0017	Conventional Hydrogen Bond
	LIG:H - MET341:O	1.9495	Conventional Hydrogen Bond
	LYS295:NZ - LIG	4.5241	Electrostatic (Pi-Cation)
	VAL281:CG1 - LIG	3.9498	Hydrophobic (Pi-Sigma)
	THR338:CG2 - LIG	3.8264	Hydrophobic (Pi-Sigma)
	LEU393:CD1 - LIG	3.3853	Hydrophobic (Pi-Sigma)
	LEU393:CD2 - LIG	3.6185	Hydrophobic (Pi-Sigma)
	MET314:SD - LIG	5.4494	Pi-Sulfur
	LIG - VAL281	4.8479	Hydrophobic (Pi-Alkyl)
	LIG - ALA293	4.1230	Hydrophobic (Pi-Alkyl)
	LIG - LEU273	4.9237	Hydrophobic (Pi-Alkyl)
	LIG - ALA293	5.3932	Hydrophobic (Pi-Alkyl)
	LIG - VAL323	5.0800	Hydrophobic (Pi-Alkyl)
	LIG - ALA403	5.0059	Hydrophobic (Pi-Alkyl)

*3-[2-(2-cyclopentyl-6-{[4-(dimethylphosphoryl)phenyl]amino}-9H-purin-9-yl)ethyl]phenol.

Table S2. Molecular interaction parameters for the binding of Isozeylanone to HSP90AA1.

Ligand	Donor-Acceptor pair	Distance (Å)	Type of Interaction
**Control*** **(NMS-E973)**	ASN51:HD21 - LIG:O	2.6894	Conventional Hydrogen Bond
	MET98:SD - LIG	4.4192	Pi-Sulfur
	PHE138 - LIG	3.7122	Hydrophobic (Pi-Pi Stacked)
	LIG:Cl - LEU107	3.8925	Hydrophobic (Alkyl)
	LIG:Cl - LEU107	4.8642	Hydrophobic (Alkyl)
	PHE138 - LIG:Cl	4.9318	Hydrophobic (Pi-Alkyl)
	PHE138 - LIG:Cl	4.6513	Hydrophobic (Pi-Alkyl)
	TYR139 - LIG:Cl	4.8866	Hydrophobic (Pi-Alkyl)
	TRP162 - LIG:Cl	4.0604	Hydrophobic (Pi-Alkyl)
	LIG - ALA55	4.7800	Hydrophobic (Pi-Alkyl)
	LIG - MET98	5.3054	Hydrophobic (Pi-Alkyl)
	LIG - LEU107	4.6120	Hydrophobic (Pi-Alkyl)
**Isozeylanone**	LIG:H - TYR139:OH	2.4353	Conventional Hydrogen Bond
	LEU107:CD1 - LIG	3.9928	Hydrophobic (Pi-Sigma)
	LEU107:CD1 - LIG	3.8577	Hydrophobic (Pi-Sigma)
	MET98:SD - LIG	3.6237	Pi-Sulfur
	PHE138 - LIG	3.6476	Hydrophobic (Pi-Pi Stacked)
	PHE138 - LIG	4.5999	Hydrophobic (Pi-Pi Stacked)
	LIG:C - MET98	5.0401	Hydrophobic (Alkyl)
	LIG:C - VAL150	3.8389	Hydrophobic (Alkyl)
	LIG:C - VAL186	5.0626	Hydrophobic (Alkyl)
	PHE138 - LIG:C	4.3454	Hydrophobic (Pi-Alkyl)
	LIG - ALA55	4.8817	Hydrophobic (Pi-Alkyl)
	LIG - ALA55	4.4188	Hydrophobic (Pi-Alkyl)
	LIG - MET98	4.3660	Hydrophobic (Pi-Alkyl)

*5-(3,4-dichloro-phenoxy)-benzene-1,3-diol.
